# Fabrication and Properties of Micro-Nanoencapsulated Phase Change Materials for Internally-Cooled Liquid Desiccant Dehumidification

**DOI:** 10.3390/nano7050096

**Published:** 2017-04-29

**Authors:** Xiaofeng Niu, Qing Xu, Yi Zhang, Yue Zhang, Yufeng Yan, Tao Liu

**Affiliations:** 1 College of Urban Construction, Nanjing Tech University, Nanjing 210009, China; allen81404009@163.com (Q.X.); yuezh25@yahoo.com (Y.Z.); 15950597352@163.com (Y.Y.); 18366188951@163.com (T.L.); 2 College of Material Science and Engineering, Anhui University of Technology, Ma’anshan 243032, China; zhy1987@ahut.edu.cn

**Keywords:** micro-nanoencapsulated *n*-octadecane, phase change properties, microstructure, compound emulsifiers, liquid desiccant

## Abstract

Micro-nanoencapsulated phase change materials (M-NEPCMs) are proposed to be useful in liquid desiccant dehumidification by restraining the temperature rise in the moisture-removal process and improving the dehumidification efficiency. In this paper, the *n*-octadecane M-NEPCMs with desirable thermal properties for internally-cooled dehumidification were fabricated by using compound emulsifiers through the in-situ polymerization method. Melamine-formaldehyde resin was used as the shell material. The effects of the mixing ratio, emulsification methods and amount of the compound emulsifiers on the morphology, size and thermal properties of the M-NEPCMs were investigated experimentally. The optimum weight mixing ratio of the compound emulsifiers is SDS (sodium dodecyl sulfate):Tween80 (polyoxyethylene sorbitan monooleate):Span80 (sorbitan monooleate) = 0.1:0.6:0.3, which achieves the best stability of the *n*-octadecane emulsion. When the compound emulsifiers are 10 wt. % of the core material, the melting enthalpy of M-NEPCMs reaches its maximum of 145.26 J/g of capsules, with an encapsulation efficiency of 62.88% and a mean diameter of 636 nm. The sub-cooling of the prepared M-NEPCMs is lower than 3 °C, with an acceptable thermal reliability after the thermal cycling test. A pre-emulsification prior to the addition of deionized water in the emulsification is beneficial to the morphology of the capsules, as the phase change enthalpy can be increased by 123.7%.

## 1. Introduction

Reducing the energy consumption of air temperature and the humidity handling process is of great significance for energy conservation. The liquid desiccant driven by low-grade heat has advantages with regards to energy saving and environmental protection [1,2,3,4]. However, the low dehumidification efficiency and the droplet carry-over problem limit the wide application of liquid desiccant technology in air conditioning systems [5,6,7,8]. The latent heat of the water vapor is released along with the moisture removal from the air and the temperature of desiccant solution rises, which leads to a decline in the water–vapor partial pressure difference between the air and the desiccant solution. Consequently, the heat and mass transfer will be greatly weakened. Therefore, an effective solution to control the temperature rise in the liquid desiccant dehumidification has become a research hotspot. Related research has shown that internally-cooled dehumidifier using coolant is an effective option to restrain the temperature rise of liquid desiccant [9]. However, the configuration of the internally-cooled dehumidifier is relatively complex and the problem of corrosion limits its application [10]. Phase change materials (PCMs) can absorb a large amount of latent heat during the phase transition from solid to liquid, while having almost no change in its temperature. It has been widely employed in many fields, including energy storage, textile fabrics and built energy systems [11,12,13,14]. However, there is no existing literature on using PCMs in a liquid desiccant system for reducing the energy consumption in air humidity handling. It is proposed that a better performance of the dehumidification process is expected by adding micro-nanoencapsulated phase change materials (M-NEPCMs) in the liquid desiccant solution to achieve a novel internally-cooled liquid desiccant dehumidification process without any configuration change in the dehumidifier. 

The M-NEPCMs with appropriate phase change temperature, good seal/microstructure characteristics and excellent thermal performance are required when it is used in the liquid desiccant dehumidifier. For the most commonly-used liquid desiccant lithium chloride (LiCl) aqueous solution, it is desirable that the temperature of the liquid desiccant solution is maintained at lower than 30 °C in the dehumidification process. The *n*-octadecane is adopted as the core material of the M-NEPCMs added into the liquid desiccant solution in this study, since its melting point (phase change temperature) is 28.2 °C and latent heat is as high as 230 J/g [15]. Due to its high mechanical toughness and strength [16], melamine-formaldehyde resin (MF) is chosen as the shell material of the M-NEPCMs for internally-cooled liquid desiccant dehumidification.

Choosing an appropriate emulsifier to prepare a stable emulsion of the core material is the basis for the fabrication of M-NEPCMs. The emulsifier plays an important role in the properties of M-NEPCMs, in particular the mean diameter and the thermal properties, which are of great importance when M-NEPCMs are applied in the liquid desiccant dehumidification. A survey of literature indicates that, because of its strong adsorbability and dispersibility in the oil-in-water emulsion system, the sodium salt of styrene–maleic anhydride copolymer (SMA) is the most commonly-used single emulsifier in the fabrication of *n*-octadecane M-NEPCMs [17,18,19,20], with only a very few researchers having adopted emulsifiers other than SMA. Su et al. [21] used styrene–maleic anhydride copolymer as a dispersant and non-ionic surfactant NP-10 as an emulsifier. Zhang and Wang tested two types of non-SMA emulsifiers in their fabrication of *n*-octadecane M-NEPCMs, including sodium dodecyl sulfate (SDS) and polyvinyl alcohol (PVA) [22]. In the fabrication of M-NEPCMs conducted by Zhan et al. [23], the non-ionic compound emulsifiers of polyoxyethylene sorbitan monooleate (Tween80) and sorbitan monooleate (Span80) were compared to the single emulsifier polyoxyethylene sorbitan monostearate (Tween60) or polyoxyethylene nonyl phenyl ether (OP-10). Luo et al. [24] compared the effects of several single emulsifiers, including Span80, Tween80, sodium dodecyl sulfonate (SDS) and cetyltrimethyl ammonium bromide (CTAB), to the mixture of Span80 and CTAB. It was found the energy storage density and the morphology of the prepared *n*-octadecane M-NEPCMs can be improved by using the compound emulsifiers [24]. Tumirah et al. adopted SDS as the emulsifier in the fabrication of *n*-octadecane microcapsules [25].

In short, the emulsifiers other than SMA have been seldom used in the preparation of *n*-octadecane M-NEPCMs in the existing literature. In this paper, we conducted a tentative study on using non-SMA emulsifiers to prepare the *n*-octadecane M-NEPCMs for applications in liquid desiccant dehumidification. The type of emulsifier and the ratio of the compound emulsifiers of SDS, Tween80 and Span80 were discussed. Furthermore, the effects of the compound emulsifiers' content on the morphology, particle size and thermal properties of the *n*-octadecane M-NEPCMs were investigated experimentally. Thermal reliability of the prepared M-NEPCMs was investigated by the thermal cycling test. In addition, the effects of two different emulsification methods were compared.

## 2. Materials and Methods

### 2.1. Materials

The capsule core material *n*-octadecane with analytical purity (≥99.7%) was purchased from Aladdin Biochemical Technology Co., Ltd. (Shanghai, China). The monomers for forming the shell, including melamine with analytical purity and formaldehyde (37 wt. % aqueous solution) were supplied by Lingfeng Chemical Reagent Company (Shanghai, China) and Xilong Chemical Co., Ltd. (Guangzhou, China), respectively. SDS used as an emulsifier was also obtained from Lingfeng Chemical Reagent Company. The other two emulsifiers tested in this study, Tween80 and Span80, were obtained from Sinopharm Chemical Reagent Co., Ltd. (Shanghai, China). Triethanolamine, ammonium chloride and acetic acid with analytical purity were used as pH control agents, being produced by Lingfeng Chemical Reagent Company, Xilong Chemical Co., Ltd. and Shanghai Shisihewei Chemical Co., Ltd. (Shanghai, China), respectively.

### 2.2. Preparation of M-NEPCMs

**Figure 1:**
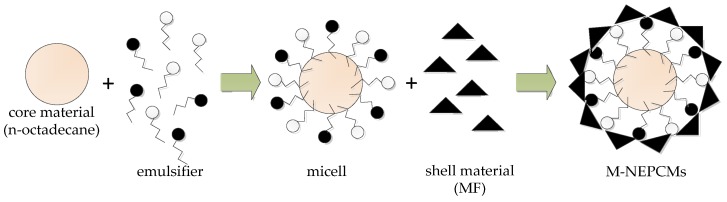
The principle of in-situ polymerization method.

The in-situ polymerization method was adopted in the preparation of M-NEPCMs [26]. The basic principle of this method is shown in Figure 1. Firstly, a stable emulsion of *n*-octadecane was prepared according to the following specific procedure: The core material *n*-octadecane (10 g) were mixed with emulsifier, before 5 mL of deionized water at a temperature of 80 °C was added in the mixture. Being immersed in a water bath at a constant temperature of 80 °C, the mixture was dispersed by a MXR-B500 high-shear homogenizer (Shanghai Muxuan Industrial Co., Ltd., Shanghai, China) at 1000 rpm for 20 min. Meanwhile, 100 mL of deionized water was added dropwise to the mixture at a rate of 5 mL per minute during the dispersion. After further dispersion for 10 min, the pH value of the emulsion was adjusted to 4.5–5.0 with acetic acid solution, before the dispersion was continued for 30 min. Finally, the oil-in-water emulsion of *n*-octadecane was obtained.

Following this, the shell of capsule was synthesized. Melamine (3 g) and formaldehyde solution (6 g, 37 wt. %) were dissolved in deionized water, before the mixture was agitated vigorously by a homogenizer (IKA Works GmbH& Co., Staufen, Germany) at the speed of 600 rpm. After 30 min, the pH value was adjusted to 8.5–9.0 with triethanolamine solution. Stirring was continued for 1 h, with a clear and transparent melamine-formaldehyde (MF) prepolymer then being obtained.

Finally, microencapsulation was conducted in a three-neck round bottomed flask equipped with a mechanical agitator. The bottom of the flask was immersed in a water bath at a temperature of 40 °C. The MF prepolymer was slowly added dropwise at a stirring speed of 400 rpm and the pH was adjusted to 5.5 with 10% ammonium chloride solution. The temperature of the water bath was raised to 65 °C and the reaction was continued for 2 h. Following this, the pH value was adjusted to 3.5–4.0 with 10% glacial acetic acid solution. The reaction was stopped after 30 more minutes of continuous stirring. The prepared samples were repeatedly washed with anhydrous ethanol and deionized water, before being filtrated to remove the impurities and unencapsulated *n*-octadecane. The purified wet M-NEPCMs were then dried in a vacuum oven (Shanghai Jinghong Laboratory Instrument Co., Ltd., Shanghai, China) at 40 °C for 24 h to obtain *n*-octadecane capsules. In this study, when 10 g of core material *n*-octadecane and 5.22 g of MF shell material were used in the preparation, finally 12–13 g of the dry M-NEPCMs can be obtained.

### 2.3. Characterization of M-NEPCMs

The surface morphology of the M-NEPCMs was observed using a SU8010 scanning electron microscope (SEM) (HITACHI, Tokyo, Japan) at an accelerating voltage of 3 kV. The tested samples were coated with a layer of gold prior to the scanning.

The phase change performance of the micro-nanocapsules was characterized by a PE Diamond differential scanning calorimeter (DSC) (PerkinElmer Instruments, Shelton, CT, USA). The DSC test was carried out from 10 to 50 °C at a ramp rate of 1 °C/min, with all measurements performed under a nitrogen protection atmosphere.

Furthermore, the diameter distribution of the micro-nanocapsules was investigated using a MS2000 laser particle size analyzer (Malvern Instruments Ltd., Malvern, UK) at a detection range of 50 nm–2500 μm. The mean diameter of the micro-nanocapsules was determined as follows:

(1) $$d_{m}=\sum_{i}d_{i}x_{i}$$

where *d*_m_ is the mean diameter of the micro-nanocapsules (nm or μm); *d_i_* is the *i*-th diameter of the micro-nanocapsules (nm or μm) and *x_i_* is the percentage of the micro-nanocapsule with particle size *d_i_* in the total number of micro-nanocapsules (%).

The encapsulation efficiency is an important index, which was defined as follow [25,27,28]:

(2) $$R=\frac{\Delta H_{m}}{\Delta H_{m,\mathrm{PCM}}}\times 100\%$$

where *R* is the encapsulation efficiency, Δ*H*_m_ is the specific melting enthalpy of M-NEPCMs (J/g of capsules and Δ*H*_m,PCM_ is the specific melting enthalpy of the pure *n*-octadecane measured by DSC (231 J/g of octadecane).

It should be noted that, Equation (2) reflects the closeness degree of the M-NEPCMs’ melting enthalpy to that of the unencapsulated pure *n*-octadecane, thus the encapsulation efficiency *R* can be used to compare the encapsulation performance of capsules prepared by different emulsifiers.

## 3. Results and Discussion

Stable and uniformly dispersed emulsion of the core material is critical for the preparation of M-NEPCMs, which has a significant impact on the capsule size and the encapsulation efficiency of capsules. Three factors affecting the stability and dispersity of the PCM emulsion, including the type and mixing ratio of compound emulsifiers, the emulsifying style as well as the amount of emulsifier, were studied and discussed as below. 

### 3.1. Mixing Ratio of the Compound Emulsifiers

According to the views of hydrophilic–lipophilic balance (HLB) [29,30], each of the emulsifiers has a numerical value indicating its hydrophilic–lipophilic balance. A HLB value of the emulsifier that is closer to the required HLB value for the emulsion dispersing system indicates a more stable prepared emulsion. 

Based on this principle, the three components of the compound emulsifiers used in this work, including SDS, Span80 and Tween80, were blended at four different ratios as listed in Table 1. These ratios were based on the consideration that the HLB value of the compound emulsifier was controlled at 10 to 13. Typically, the HLB value required for oil-in-water emulsion is in the range of 8 to 18. The HLB value of the compound emulsifier can be obtained approximately by the following equation:

(3) $$HLB_{\mathrm{com}}=\xi_{\mathrm{SD}}HLB_{\mathrm{SD}}+\xi_{\mathrm{SP}}HLB_{\mathrm{SP}}+\xi_{\mathrm{TW}}HLB_{\mathrm{TW}}$$

where, *HLB* is the hydrophilic–lipophilic balance value; *ξ* is the weight ratio of a single emulsifier in the compound emulsifiers; the subscript com, SD, SP and TW represents the compound emulsifiers, SDS, Span80 and Tween80, respectively.

The compound emulsifiers with different mixing ratios were tested respectively to prepare an emulsion of *n*-octadecane. The preparation conditions were all the same, except for the mixing ratios of the emulsifiers. The total amount of the compound emulsifier was 15% of the mass of *n*-octadecane. After emulsification at a constant temperature of 80 °C by the homogenizer, the four emulsion samples prepared from compound emulsifiers with four different mixing ratios were placed in beakers for 2 h. When the emulsification just finished, all the four emulsions were uniformly dispersed. The stability of them was continuously observed by the naked eye during 2 h standing.

**Figure 2:**
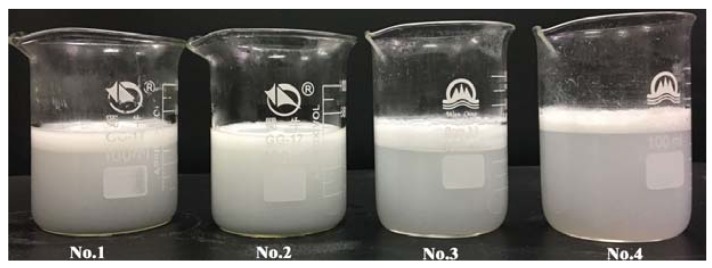
Images of four samples from different mixing ratios of emulsifiers after 2 h standing.

The stability of the four emulsion samples after two hours standing are shown in the Figure 2, with the codes from left to wright corresponding to the sample codes listed in the Table 1. As can be seen from the figure, Sample 2 is dispersed most uniformly without significant phase separation. However, obvious delamination is observed in the other three samples. The phenomenon shown in Figure 2 indicates that the stability of emulsion in sample two is the best among the four samples. In short, Sample 2 contains the optimal mixing ratios of compound emulsifiers used for emulsion. 

It can be deduced that the best stability of the emulsion in Sample 2 may be attributed to the HLB value of the used compound emulsifiers. By adjusting the mixing ratios of the three components in the compound emulsifiers to *ξ*_SD_:*ξ*_SP_:*ξ*_TW_ = 0.1:0.6:0.3, the HLB value of the compound emulsifiers (*HLB*_com_ = 11.08) can reach or come very close to the required value of the *n*-octadecane emulsion system prepared in this study. In comparison, the HLB values obtained from other compounding ratios are relatively large compared to that required for the emulsion system. 

**Figure 3:**
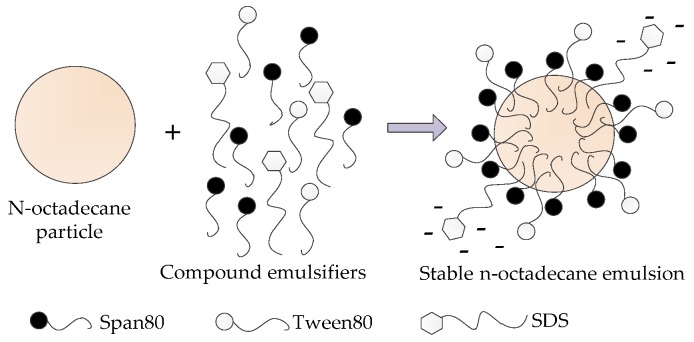
Emulsification mechanism of the compound emulsifiers.

The emulsification mechanism of the composite emulsifiers used herein can be explicated with reference to Figure 3. The HLB value of Span80 is relatively small, thus its lipophilicity is higher and results in the arrangement of Span80 molecules on the interfacial film that is closer to the oil phase. Essentially, this arrangement causes the Span80 molecules to be closer to the thin droplets of the core material *n*-octadecane. In comparison, the HLB value of Tween80 is higher and its hydrophilicity is higher, thus resulting in its arrangement on the interface film being closer to the water phase. The molecules of Span80 and Tween80 are arranged at intervals on the liquid film surface, which makes the emulsifier molecules on the interface film more closely aligned and thereby enhances the stability of the emulsion. Moreover, the anionic emulsifier SDS has an HLB value of 40 and its high hydrophilicity can effectively reduce the surface tension. Furthermore, the surface-active anions form a negative electric field on the surface of the core particle. This electric repulsive force among the particles and the increase in zeta-potential of the particles results in the particles being more difficult to agglomerate, again improving the stability of the emulsion. 

### 3.2. Effect of Emulsification Method on M-NEPCMs Properties

According to the preparation procedures for M-NEPCMs described in Section 2.1. and the aforementioned optimal mixing ratio of the compound emulsifiers (*ξ*_SD_:*ξ*_SP_:*ξ*_TW_ = 0.1:0.6:0.3), 10 g of *n*-octadecane, 1.5 g of the compound emulsifiers and 5 mL of the deionized water at 80 °C were first mixed, before two emulsification methods were tested respectively in the subsequent emulsification process. The details of the two emulsification methods are listed in Table 2.

As shown in the Table 2, the only difference between the two emulsification methods is a pre-emulsification in Method (B). All the other conditions, including the volume of deionized water added, the dropping rate of deionized water, the stirring speed as well as the temperature of emulsification, are all the same. In Method (A), the emulsification by the homogenizer and the dropping of deionized water are conducted simultaneously. In comparison, the mixture is first pre-emulsified for 5 min by the homogenizer in Method (B), before the deionized water is added dropwise to the mixture when the emulsification is continued.

**Figure 4:**
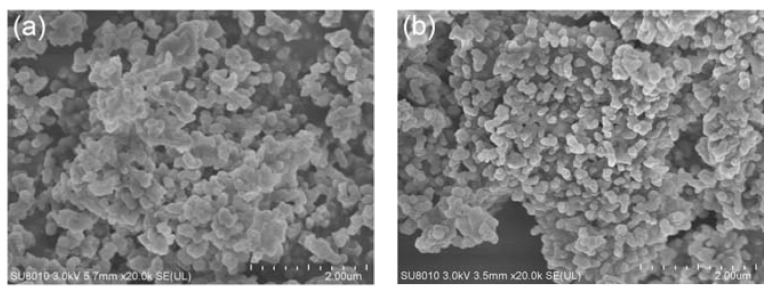
SEM (scanning electron microscope) images of M-NEPCMs (Micro-nanoencapsulated phase change materials) prepared with different emulsification Methods: (**a**) Method A and (**b**) Method B.

**Figure 5:**
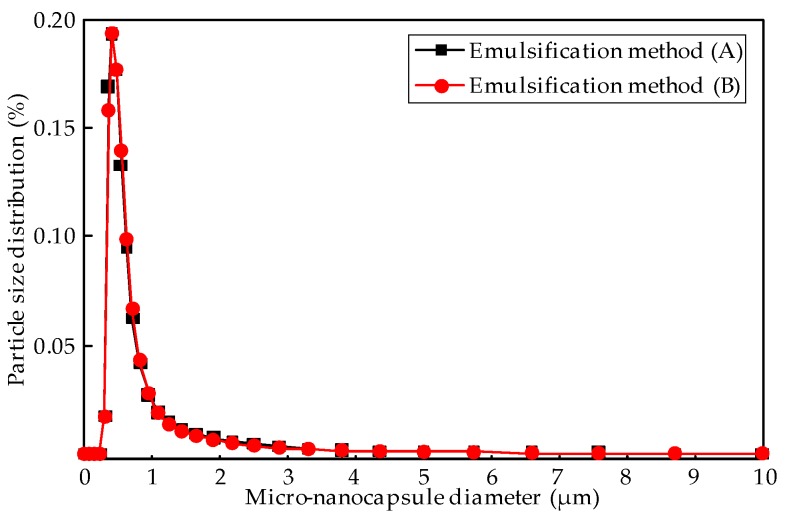
Particle size distribution of M-NEPCMs prepared with emulsification Methods (A) and (B).

The morphology of M-NEPCMs prepared using the two emulsifier methods is shown in Figure 4. As seen in the figure, compared with the M-NEPCMs prepared with Method (A), the M-NEPCMs in Figure 4b have more regular spherical shape, smoother surfaces and better dispersibility, while the micro-nanocapsules in Figure 4a show an obvious agglomeration. Therefore, the morphology of micro-nanocapsules prepared with Method (B) is better than that of Method (A). Figure 4 also shows that the M-NEPCMs have a similar size when prepared with either of the two methods, which is further proved by the particle distribution of the micro-nanocapsules shown in Figure 5. There was almost no difference in the distributions of the particles with the same diameters obtained by different emulsification methods. Furthermore, the mean diameters obtained from Equation (1) shows that the mean diameter of the micro-nanocapsules prepared with emulsification Method (A) is 630 nm, while that of the micro-nanocapsules prepared by Method (B) is 632 nm. Therefore, the two emulsification methods tested in this paper have little difference with regards to the size distribution of micro-nanocapsules.

As shown in Table 3, the phase change temperatures of the M-NEPCMs prepared by the two emulsification methods are very close. However, the phase change enthalpy of the M-NEPCMs obtained by using the emulsification Method (A) was relatively lower, with its melting enthalpy and crystallization enthalpy being 61.27 J/g of capsules and 70.56 J/g of capsules, respectively. The corresponding encapsulation efficiency of micro-nanocapsules is 26.52%. In comparison, the phase change enthalpy of the micro-nanocapsules prepared by using the emulsification Method (B) is higher, with the melting enthalpy and the crystallization enthalpy reaching 137.07 J/g of capsules and 137.86 J/g of capsules, respectively. Its corresponding encapsulation efficiency is 59.34%. The melting enthalpy of emulsification Method (B) is 123.7% higher than that of Method (A). Therefore, the two emulsification methods have different influences on the thermal properties of the prepared M-NEPCMs. Compared with the emulsification Method (A), the phase change enthalpy of M-NEPCMs can be improved significantly by Method (B), which may be attributed to the high stability and dispersity of the emulsion prepared using Method (B). As previously mentioned, the only difference between the two emulsification methods is the 5 minutes of pre-emulsification prior to the addition of deionized water in emulsification process (B), which may have an impact on the thermal properties of the M-NEPCMs as follows:

For the emulsification Method (A), deionized water is added simultaneously at the beginning of emulsification. Under the shearing force by the stirring of the homogenizer, the continuous addition of deionized water will reduce the effective shearing forces acting on the capsule core material *n*-octadecane, which results in the deterioration of the emulsification of the core material. Thus, the encapsulation efficiency will be decreased in the subsequent microencapsulation process. Correspondingly, the phase change enthalpy of the prepared M-NEPCMs will be relatively low. 

In the case of using emulsification Method (B) in the emulsification, the deionized water is not added until the emulsification is carried out for 5 minutes. In the 5 minutes of pre-emulsification, the core material was sufficiently emulsified. Thus, the deionized water added afterwards will not affect the stability of the emulsion, which leads to a higher encapsulation efficiency during microencapsulation and a higher phase change enthalpy of the prepared MEPCM.

In addition, as a result of the pre-emulsification in emulsification Method (B), the emulsifier molecules can be fully and uniformly dispersed on the surface of the water droplets, thus resulting in the fine aqueous droplets being covered with the emulsifier molecules to form a stable interface film. When the deionized water is added gradually thereafter, the continuous phase will be aqueous under the shearing action and a complete phase inversion will occur, with the dispersed phase (core material) being completely coated by the interfacial film to form an emulsion with high stability. For emulsification Method (A), simultaneous addition of deionized water at the beginning of the emulsification results in a partial coverage of the emulsifier with the fine aqueous droplets. Consequently, the incomplete phase inversion from the oil continuous phase to the aqueous continuous phase will occur, which finally leads to the lower stability of the *n*-octadecane emulsion. Therefore, the stability of the *n*-octadecane emulsion prepared by emulsification Method (B) is better, with the micro-nanocapsules prepared by emulsion Method (B) having a higher enthalpy of phase change. The stability of the core material emulsion prepared using emulsion Method (A) is relatively poor and hence, the phase change enthalpy of micro-nanocapsule obtained from Method (A) is also low.

### 3.3. Effect of Compound Emulsifiers Amount on M-NEPCMs Properties

Due to the thermal properties of the micro-nanocapsules prepared by emulsification Method (B) being obviously superior to those of Method (A), the following experiments in this present student were based on emulsification Method (B). The effects of the amount of compound emulsifiers on the morphology, size distribution and thermal properties of the M-NEPCMs were investigated. The amount of emulsifier varied from 5% to 25% of the core material content at intervals of 5%. The detailed components of the M-NEPCMs are listed in Table 4. It should be noted that the mixing ratio of the compound emulsifiers is fixed to the optimal value (*ξ*_SD_:*ξ*_SP_:*ξ*_TW_ = 0.1:0.6:0.3) obtained in Section 3.1.

#### 3.3.1. Effect of Emulsifier Amount on Morphology of M-NEPCMs 

**Figure 6:**
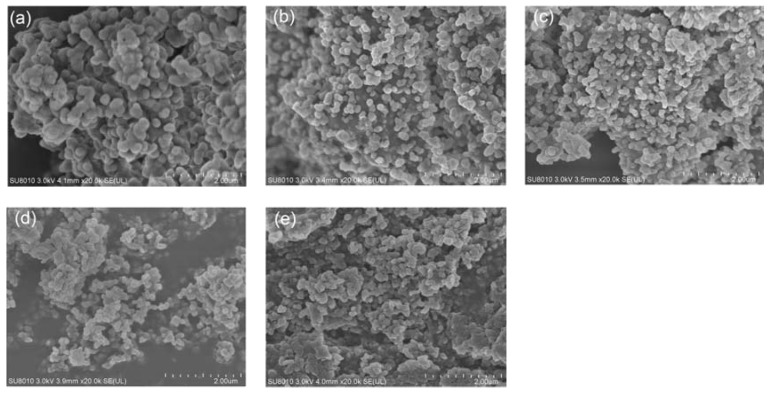
SEM images of micro-nanocapsules prepared with different amounts of emulsifier: (**a**) Sample 1, *R*_EC_ = 5%; (**b**) Sample 2, *R*_EC_ = 10%; (**c**) Sample 3, *R*_EC_ = 15%; (**d**) Sample 4, *R*_EC_ = 20%; (**e**) Sample 5, *R*_EC_ = 25% (*R*_EC_ is the ratio of the mass of emulsifier to that of the core material (%)).

The SEM images of Samples 1 to 5 are shown in Figure 6. It can be found that the dispersibility of Samples 4 and 5 was lower than that of other samples, while the micro-nanocapsules of Sample 4 and Sample 5 show an irregular agglomeration. Furthermore, the micro-nanocapsules of Sample 1 and Sample 3 have slight agglomeration. The morphology of micro-nanocapsules of Sample 2 is relatively better compared to the other four samples. 

The images shown in Figure 6 indicate that with an increase in the amount of emulsifier, the dispersity of micro-nanocapsule particles first increases and then drops, which is attributed to the variation in the surface tension of the emulsion along with an increase in the amount of emulsifier. When the content of emulsifier is increased, the surface tension of the *n*-octadecane emulsion first drops, before the intermolecular arrangement of the adsorbed emulsifier molecules on the interfacial film becomes more and more tight. Thus, the interfacial film strength and the emulsion stability are enhanced, which results in the improvement in the dispersibility of the prepared micro-nanocapsules. While the critical micelle concentration (CMC) of the compound emulsifiers can be reached with a continuous increase in the amount of emulsifier, the emulsifier molecules on the interfacial film will be saturated, which means the *n*-octadecane emulsion reaches its maximum stability. When the concentration of emulsifier increases further, the surface tension of the emulsion will not decrease any more, the emulsifier molecules adsorbed on the water–PCM interface film becomes supersaturated and spontaneous agglomeration can easily occur among the excessive emulsifier molecules in the emulsion. Finally, this leads to an aggravation of agglomeration among the micro-nanocapsules.

#### 3.3.2. Effect of Emulsifier Amount on Size Distribution of M-NEPCMs

**Figure 7:**
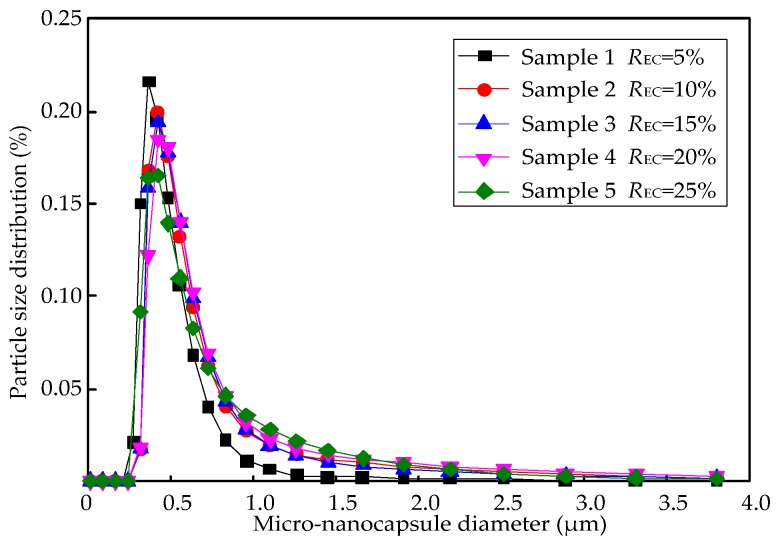
Particle size distribution of micro-nanocapsules prepared by different amounts of emulsifier.

Figure 7 shows the particle size distribution of the M-NEPCMs prepared with different amounts of emulsifier. The particle size of the Samples 1 to 5 is mostly concentrated in the range of 300 nm to 1000 nm, with the ratio of the particle diameter of about 500 nm being the highest. Furthermore, it can be seen in the figure that the particle size distribution becomes more and more varied with an increase in the amount of emulsifier.

The mean diameter of each sample was calculated by Equation (1), as shown in Table 5. Compared with the data in literature (see Section 3.3.3), the M-NEPCMs prepared in this study are much smaller, which is beneficial for the cycling of M-NEPCMs in the liquid desiccant system. The mean diameter of the micro-nanocapsules increased from 484 to 723 nm as the amount of the emulsifier increased from 5% to 25% of the core material content. This is mainly because the viscosity of the emulsion gradually increases with an increase in the amount of emulsifier, while the stirring speed of emulsification was maintained at 1000 rpm. Therefore, the shearing forces acting on the core material were weakened, so the droplet diameter of the core material in the emulsion was increased, which results in the subsequently obtained micro-nanocapsule size also showing a slight increase.

#### 3.3.3. Effect of Emulsifier Amount on Thermal Properties of M-NEPCMs

**Figure 8:**
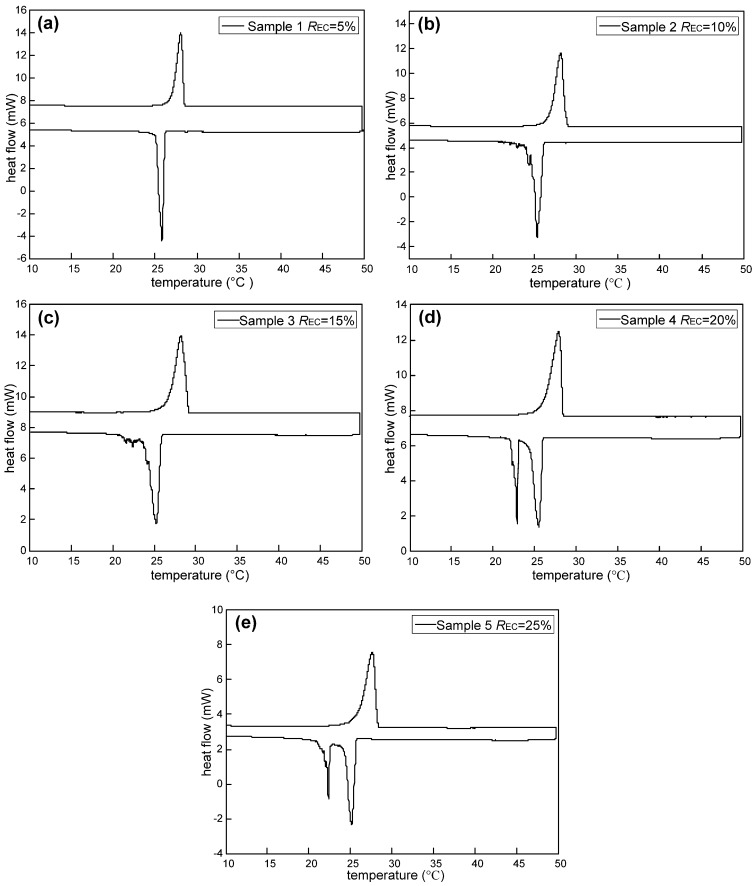
DSC (differential scanning calorimeter) thermograms of the M-NEPCMs prepared with different amounts of emulsifier.

The thermal properties of M-NEPCMs synthesized by using different amounts of emulsifier, including the phase change enthalpies and phase change temperatures, were measured by DSC, with the heating and cooling thermograms shown in Figure 8. The upper curve in the figure depicts the heating thermograms, while the lower curve depicts the cooling thermograms. From the DSC heating thermograms, a single peak can be visibly observed for all the samples. For the cooling thermograms, there is a single trough for both Sample 1 and Sample 2, while double troughs are shown in the other three samples. Figure 8 shows that the melting temperatures and the crystallization temperatures of each sample are close to each other, which is supported by the detailed data listed in Table 6.

As shown in Table 6, the phase change enthalpy of Sample 2 is the highest among the tested samples, with a melting enthalpy of 145.26 J/g of capsules and a crystallization enthalpy of 147.01 J/g of capsules. The corresponding encapsulation efficiency of Sample 2 reached almost 63%. Therefore, for the compound emulsifiers (*ξ*_SD_:*ξ*_SP_:*ξ*_TW_ = 0.1:0.6:0.3) used in the preparation of *n*-octadecane M-NEPCMs, the most suitable amount of emulsifier is 10% of the mass of the core material in order to reach the highest phase change enthalpy of M-NEPCMs. Furthermore, the melting enthalpy of Sample 2 is only 1.24 J/g of capsules lower than the highest value (146.5 J/g of capsules) from the literature (Table 7). However, the size of Sample 2 is 0.636 μm, which is much smaller than the average size in literature [22]. Therefore, the comprehensive performance of the M-NEPCMs prepared in this study is better than that in the literature. 

Moreover, the phase change enthalpy of M-NEPCMs increases with an increasing amount of emulsifier and then decreases, which can be explained as follows: A small increase in the amount of emulsifier is beneficial for decreasing the surface tension of the *n*-octadecane emulsion as well as gradually enhancing the strength of *n*-octadecane-water interfacial film and the emulsion stability. This results in an improvement in the encapsulation efficiency and more core materials are encapsulated by the MF shell material, thus explaining the increasing tendency in the phase change enthalpy of the prepared M-NEPCMs. With a continuous increase in the amount of emulsifier, the supersaturated emulsifier molecules on the interfacial film will be agglomerated, which leads to an increase in the emulsion viscosity. Therefore, the encapsulation efficiency will decrease and correspondingly, the phase change enthalpy of the prepared M-NEPCMs will decrease gradually. 

The phase change temperature of the M-NEPCMs is also critical for its application in the internally-cooled liquid desiccant system. Figure 7 and Table 6 shows that all the melting temperatures of the tested samples were in the range of 25.9–29.1 °C, which is in accordance with the required temperature range of desiccant solutions in the dehumidification process. In addition, the five samples have the subcooling temperature of 2.24 to 2.96 °C. This low subcooling temperature indicates that even though the mean diameters of the samples are smaller than 1 μm, relatively large amounts of core material *n*-octadecane were still encapsulated in the capsules, i.e., the encapsulation efficiency is relatively high for the M-NEPCMs fabricated using the compound emulsifiers in this study. Furthermore, the crystallization temperature of the samples ranges from about 20–26.1 °C. This means that naturally cold sources (such as the water and air) can be employed for the cooling in re-solidification of the M-NEPCMs and hence, this could result in a reduction in cooling energy consumption. 

### 3.4. Thermal Cycling Test of the Prepared M-NEPCMs

When the *n*-octadecane M-NEPCMs is used in the liquid desiccant system, it will be cycled at a temperature range between 20 and 70 °C. The M-NEPCMs should maintain stable thermal properties for long-term cycling at the working condition of fluctuating temperatures. Therefore, a thermal cycling test was performed to evaluate the thermal reliability of the prepared M-NEPCMs in this study. The M-NEPCMs of Sample 4 in Table 6 was chosen as the tested sample. Two grams of the sample was loaded in a glass vial, before being heated at 80 °C and cooled at 10 °C using two water-baths alternately. The procedure was performed consecutively for 300 times. The changes in the thermal properties and morphology of the sample after thermal cycling were measured by DSC and SEM, respectively. 

**Figure 9:**
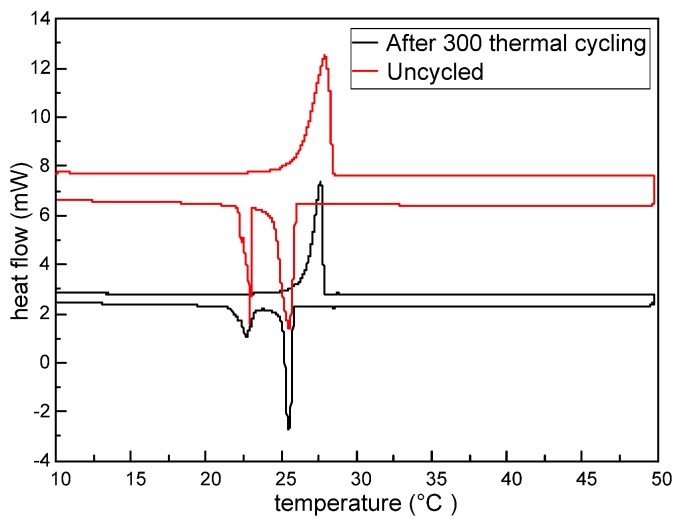
DSC thermograms of the M-NEPCMs before and after thermal cycling 300 times.

The DSC thermograms of the sample before and after thermal cycling 300 times are shown in Figure 9, with its thermal properties given in Table 8. As observed from Figure 9, after thermal cycling 300 times, the DSC heating and cooling curves are basically consistent with those before the thermal cycling. The detailed thermal properties listed in Table 8 show that the peak melting temperature of the tested M-NEPCMs changes by 0.27 °C, while the two peak crystallization temperatures change by 0.03 and 0.22 °C. As for the enthalpy of phase change after thermal cycling 300 times, the melting enthalpy of the tested M-NEPCMs changes by 2.09 J/g of capsules and the crystallization enthalpy changes by 1.95 J/g of capsules. These changes in phase change temperatures and phase change enthalpy of the M-NEPCMs with repeated thermal cycling are acceptable for its application in the liquid desiccant system. Thus, the test results prove the high thermal reliability of the M-NEPCMs prepared by using the compound emulsifiers in this study.

**Figure 10:**
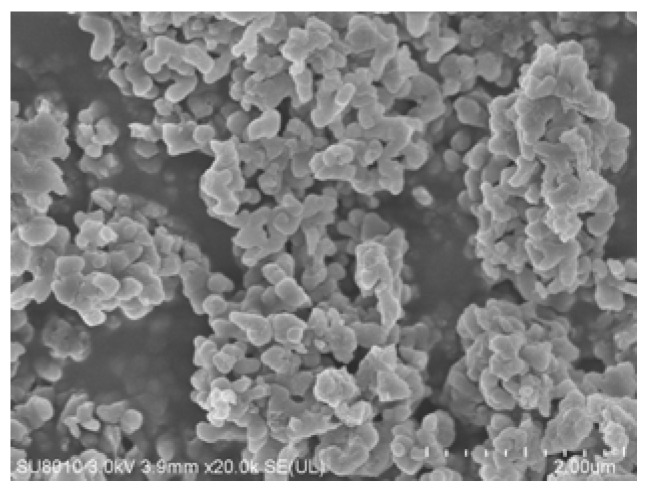
SEM images of M-NEPCMs after thermal cycling 300 times.

In addition, the SEM image of the M-NEPCMs after the thermal cycling test is shown in Figure 10. Compared with its SEM image before the thermal cycling (see Figure 6d), it can be observed that the changes in the morphology and shape of the capsule particles after thermal cycling 300 times are not significant. Moreover, no obvious rupture of the capsules and leakage of *n*-octadecane are observed in the figure, which indicates that repeated heating and cooling cycles has little influence on the structure and mechanical stability of the prepared M-NEPCMs.

## 4. Conclusions

In this paper, the compound emulsifiers of non-ionic emulsifiers Tween80 and Span80 as well as the anionic emulsifier SDS were used in the fabrication of the *n*-octadecane M-NEPCMs through the in-situ polymerization method. The effects of the mixing ratio and amount of the compound emulsifiers on the stability of emulsion, the morphology, particle size and the thermal properties of the M-NEPCMs were investigated experimentally. The main findings are as follows:

(1) When the weight mixing ratio of the compound emulsifiers is *ξ*_SD_:*ξ*_SP_:*ξ*_TW_ = 0.1:0.6:0.3, maximum stability of the *n*-octadecane emulsion is reached. The phase change temperatures of the M-NEPCMs prepared by using this mixing ratio of compound emulsifiers can match the requirements for the internally-cooled liquid desiccant system. The melting temperatures of the prepared M-NEPCMs are from 25.9 to 29.1 °C and its subcooling temperature is lower than 3 °C. Furthermore, the thermal properties changes of the M-NEPCMs after thermal cycling 300 times are relatively small and acceptable.

(2) With an increase in the amount of emulsifier from 5% to 25% of the mass of the capsule core material, the phase change enthalpy of the prepared M-NEPCMs first increases and then decreases, while the mean diameter of the micro-nanocapsules increases from 484 to 723 nm. When the content of emulsifier is 10% of the mass of capsule core material, the phase change enthalpy reaches its highest value of 145.26 J/g of capsules, with an encapsulation efficiency of 62.88% and a mean size of the micro-nanocapsules being as small as 636 nm on diameter. 

(3) The comparison of two emulsification methods shows that the melting enthalpy of the M-NEPCMs obtained by using emulsification Method (B) is 123.7% higher than that from Method (A). Moreover, the morphology of micro-nanocapsules prepared with Method (B) is better than that of Method (A). The only difference between the two emulsification methods is five minutes of pre-emulsification prior to the addition of deionized water in Method (B). However, the M-NEPCMs from the two emulsification methods have little difference with regards to the size distribution. 

## Figures and Tables

**Table 1:** HLB (hydrophilic–lipophilic balance) values of the compound emulsifiers with different mixing ratios.

Emulsion No.	*ξ*_SD_	*ξ*_SP_	*ξ*_TW_	*HLB*_com_
1	0.07	0.62	0.31	10.12
2	0.10	0.60	0.30	11.08
3	0.13	0.58	0.29	12.04
4	0.16	0.56	0.28	13.01

**Table 2:** Details of the two emulsification methods.

Emulsification Methods	Method (A)	Method (B)
Pre-Emulsification (min)	0	5
Volume of Deionized Water added (mL)	100	100
Dropping Rate of Deionized Water (mL/min)	5	5
Stirring Speed (rpm)	1000	1000
Temperature of Emulsification (°C)	80	80

**Table 3:** Thermal properties of M-NEPCMs prepared with different emulsification methods.

Emulsification Methods	Method (A)	Method (B)
Onset Melting Temperature *T*_om_ (°C)	25.11	26.76
Peak Melting Temperature *T*_pm_ (°C)	28.38	28.22
Onset Crystallization Temperature *T*_oc_ (°C)	25.12	25.84
Peak Crystallization Temperature *T*_pc_ (°C)	23.4	25.26
Melting Enthalpy Δ*H*_m_ (J/g of capsules)	61.27	137.07
Crystallization Enthalpy Δ*H*_c_ (J/g of capsules)	70.56	137.86
Encapsulation Efficiency *R* (%)	26.52	59.34

**Table 4:** Components of M-NEPCMs prepared with different amounts of emulsifier.

Sample No.	*m*_core_ (g)	*m*_em_ (g)	*m*_MF_^1^ (g)
1	10.0	0.5	9.0
2	10.0	1.0	9.0
3	10.0	1.5	9.0
4	10.0	2.0	9.0
5	10.0	2.5	9.0

^1^ *m*_core_, *m*_em_, *m*_MF_ is the mass of the core material, shell material and the emulsifier, respectively.

**Table 5:** Mean diameter of capsules prepared by different amounts of emulsifier.

Sample No.	*R*_EC_ (%)	*d*_m_ (μm)
1	5	0.484
2	10	0.636
3	15	0.630
4	20	0.684
5	25	0.723

**Table 6:** Thermal properties of M-NEPCMs prepared by using different amounts of emulsifier.

Sample No.	1	2	3	4	5
Ratio of the Mass of Emulsifier to that of the Core Material *R*_EC_ (%)	5	10	15	20	25
Onset Melting Temperature *T*_om_ (°C)	27.00	26.81	26.76	26.12	25.97
Peak Melting Temperature *T*_pm_ (°C)	28.06	28.14	28.22	27.88	27.62
Onset Crystallization Temperature *T*_oc_ (°C)	26.13	26.01	25.84	25.96	25.65
Peak Crystallization Temperature *T*_pc_ (°C)	25.82	25.32	25.26	25.51	25.15
Melting Enthalpy Δ*H*_m_ (J/g of capsules)	137.62	145.26	137.07	117.12	111.36
Crystallization Enthalpy Δ*H*_c_ (J/g of capsules)	137.93	147.01	137.86	115.51	109.78
Encapsulation Efficiency *R* (%)	59.58	62.88	59.34	50.70	48.21

**Table 7:** The size and melting enthalpy of *n*-octadecane M-NEPCMs in the literature.

Ref.	Emulsifier	Mean Particle Size (μm)	Melting Enthalpy (J/g of capsules)
[22]	SDS ^1^	8.93	111.5
[22]	SMA ^2^	14.76	146.5
[23]	OP-10 ^3^	5.34 ± 0.14	130.6 ± 4.3
[23]	Tween60 ^4^	3.71 ± 0.07	143.1 ± 2.2
[23]	Tween80 ^5^ & Span80 ^6^	3.46 ± 0.08	111.3 ± 1.9
[24]	CTAB ^7^	20	48
[24]	Span80 ^6^	10–15	62
[24]	SDS ^1^	30–40	25
[24]	Tween80 ^5^	5–10	12.5
[24]	Span80 ^6^ & CTAB ^7^	20	70
[25]	SDS ^1^	0.127 ± 0.03	117.3

^1^ sodium dodecyl sulfate, ^2^ sodium salt of styrene–maleic anhydride copolymer, ^3^ polyoxyethylene nonyl phenyl ether, ^4^ polyoxyethylene sorbitan monostearate, ^5^ polyoxyethylene sorbitan monooleate, ^6^ sorbitan monooleate, ^7^ cetyltrimethyl ammonium bromide.

**Table 8:** Thermal properties of M-NEPCMs (Sample 4) before and after thermal cycling 300 times.

Times of Thermal Cycling	Melting			Crystallization			
	*T*_om_ (°C)	*T*_pm_ (°C)	*ΔH*_m_ (J/g of Capsules)	*T*_oc_ (°C)	*T*_pc_ (°C)		*ΔH*_c_ (J/g of Capsules)
0	26.12	27.88	117.12	25.96	25.51	22.90	115.51
300	26.63	27.61	115.03	25.81	25.48	22.68	113.56
